# Research on consumers’ purchase intention of cultural and creative products—Metaphor design based on traditional cultural symbols

**DOI:** 10.1371/journal.pone.0301678

**Published:** 2024-05-13

**Authors:** Lili Liu, Hongxia Zhao

**Affiliations:** College of Economics and Management, Zhengzhou University of Light Industry, Zhengzhou, China; COMSATS University Islamabad, PAKISTAN

## Abstract

Chinese traditional cultural symbols possess great aesthetic and cultural value, and are widely utilized in product design. In this study, we explore the relationship between metaphor design based on traditional cultural symbols, customer experience and cultural identity, and further estimate how these three variables stimulate consumers’ perceived value to generate consumers’ purchase intention. Based on existing traditional cultural literature and Stimulus-organism-response theory (SOR), we proposed a theoretical research model to characterize the relationship among metaphor design based on traditional cultural symbols, customer experience, cultural identity, perceived value and consumers’ purchase intention. A research survey was conducted and 262 questionnaires were collected in total with 241 valid. We used Smart PLS graph version 3.0 for data analysis. Results indicate that the cognition of metaphor design based on traditional cultural symbols and customer experience has a direct and significant impact on the emotional value thereby, eliciting consumers’ purchase intention, metaphor design based on traditional cultural symbols is directly and indirectly (i.e., through customer experience or perceived value) positively associated with consumers’ purchase intention, also customer experience is directly and indirectly (i.e., through perceived value) associated with consumer purchase intention, cultural identity mediates the indirect effect of customer experience and perceived value on purchase intention, the moderating role of cultural identity between customer experience and perceived value is not significant. Our findings help to expand the existing literature on consumer purchase intentions by rationally using traditional cultural symbols in the product metaphor design.

## Introduction

The cultural and creative product industry in China is experiencing rapid expansion, accompanied by a growing demand for culture. The primary focus of this paper is on cultural and creative products that are conceived around traditional cultural symbols, notably emblematic Chinese motifs such as dragons and phoenixes, which are skillfully incorporated into contemporary design paradigms, thereby imbuing these artifacts with cultural significance. Examples of such products encompass tea sets, tea gift boxes, calligraphy pen and ink sets, as well as mobile phone cases and related accessories. Therefore, following the growing impact of culture on society and economy, cultural and creative products, commonly known as cultural and creative industries products, are artifacts that revolve around culture and creativity, deriving unique value from distinctive design, expression, or production. It now serves as a key engine for GDP expansion in a number of nations and is important in the struggle for cultural soft power on the international stage. The "Fourteenth Five-Year Plan" and the long-term objective of 2035 both present a comprehensive framework for the contemporary cultural industry [1]. Under the collaborative efforts and support from various governmental departments and the broader societal community, the cultural and creative industries have experienced noteworthy growth. Notably, creative products characterized by regional cultural nuances have emerged as novel favorites among consumers. From a market perspective, cultural creativity has disrupted the traditional design and production methods of products, becoming a means to enhance their economic benefits and competitiveness. To respond to changing market demands and consumer preferences, a fusion of traditional culture and modern design is essential.

In today’s globally connected world, communication between countries is more frequent, which promotes cultures integration. Traditional Chinese culture embodies a rich array of meanings and ideals, encompassing writing, artwork, patterns, hues, architecture, and music that have evolved within Oriental culture. Chinese traditional symbols are perceived and used as distinguishing features with particular cultural connotations. They maintain their conceptual significance and aesthetic qualities while encompassing value, heritage, symbolism, decoration, and function. A "National Tide" of culturally distinctive items has resulted from the economic market’s combination of cutting-edge industries with exceptional traditional Chinese culture [2]. A few examples of cultural and creative goods produced in the Forbidden City are the "Auspicious Beast Opera Palace" scented candles, the "A Thousand Miles of Rivers and Mountains" paper carving lamp, the Forbidden City wax seal, and the Flower God’s headband [3]. By incorporating historical and cultural components into innovative product design, products create awareness and appreciation for these elements, thereby increasing consumer demand. Since cultural and creative products are essentially commodities, their long-term viability ultimately depends on whether consumers intend to buy them. Accurately positioning customer preferences to satisfy rising consumer demand is a significant difficulty in the study and production of cultural and creative products. Although earlier research has shown that product design has an impact on consumers’ purchase decisions, there haven’t been any empirical studies on the use of traditional cultural symbols in product design. Previous research has predominantly focused on consumers’ willingness to purchase cultural and creative products from institutions such as the Palace Museum and marine cultural tourism. However, the exploration of metaphor design application in cultural and creative products remains limited, particularly in the context of products designed with metaphors based on traditional Chinese cultural symbols. Therefore, this study takes a novel perspective, aiming to investigate the factors influencing consumers’ willingness to purchase cultural and creative products. Given the scarcity of in-depth examinations, especially regarding metaphor design based on traditional cultural symbols, a comprehensive exploration of metaphorical design in traditional cultural symbols becomes imperative for effectively shaping consumers’ purchasing intentions. The significance of this study is underscored by several key points. Firstly, culturally innovative products designed with traditional symbols play a pivotal role in the market, necessitating an understanding of consumer purchasing intentions to gauge market demands. Secondly, the research delves into an in-depth exploration of how cultural and creative products, rooted in traditional symbols, can concurrently preserve traditional culture while embracing innovation, thereby harmonizing the cultural relationship between tradition and modernity. Thirdly, by focusing on consumer psychology and behavior, the study aims to elucidate factors related to cultural symbols, providing valuable insights for market promotion and product design recommendations. Additionally, insights into consumer purchasing intentions are instrumental for companies to optimize marketing strategies, enhance product competitiveness, and contribute to the sustainable development of the cultural and creative industries. In conclusion, this research holds positive implications for a comprehensive understanding of the dynamic cultural and creative market, offering theoretical and practical support for the robust development of the cultural and creative industries.

In conclusion, this paper examines the impact of traditional cultural symbol product design on consumer purchase intentions by exploring dominant and recessive metaphors, customer experience, cultural identity, and perceived value across different dimensions. According to the relationship between the measured variables, a theoretical model is established. We investigate the many pathways and mechanisms with which product metaphor design of conventional cultural symbols and customer experience affect consumers’ perceived value and buy inclinations through questionnaire surveys and empirical tests of structural equations. Our research contributes to the existing body of knowledge in three key ways. Firstly, it expands the application of customer experience theory, perceived value theory, and the SOR model to new sectors, providing a theoretical foundation for the metaphor design mechanism of traditional cultural symbols. Secondly, this study empirically examines product metaphor theories, addressing some of the limitations of earlier research by analyzing current research theories and addressing research gaps. Thirdly, our findings can be utilized to evaluate metaphor patterns and designs, apply them to the preservation and propagation of traditional culture, and generate innovative ideas for product development and design.

The remaining sections of the paper are organized as follows: The literature review section provides a review of relevant literature. In theoretical basis and research hypothesis section, we establish the theoretical model and examine the influence mechanism between the measured variables. Research process section introduces the questionnaire design and provides a description of the data. The analysis of the model results, encompassing the common method deviation test, the reliability and validity test of the measurement model, and the structural model test, is carried out in data analysis section. Discussion section is dedicated to the discussion, and last section addresses the research findings and their management implications.

## Literature review

### Metaphor design of traditional cultural symbols

The incorporation of traditional culture into modern design has steadily gained prominence as cultural diversity has grown. Traditional cultural icons are frequently used in metaphorical designs [4]. Designers can add cultural connotation and particular cultural charm to their works by reinterpreting and utilizing traditional cultural symbols. Metaphor can be a useful tool for designers in the realm of product design, both throughout the design process and in the actual products themselves. Metaphor can be used to reframe and address design issues. Users can learn to personify and orient products through the use of metaphors. The dominant metaphor is the metaphor of nature and artifact, whereas the recessive metaphor is characterized by symbolic culture and reveals the function of metaphor. Previous studies have split metaphorical qualities into dominant metaphor and recessive metaphor. In their study of dominant and recessive metaphor, Taylor [5] looks at the words high and tall, which are nearly synonyms, and makes the case that they have different meanings when referring to verticality. It is suggested that the co-extension connection, with high identifying the dominant perspective and tall the recessive vantage, might be used to insightfully examine the distribution of English adjectives. Readers are more likely to remember dominant metaphors than recessive ones, according to research on the cognitive processing of dominant and recessive metaphor in literary texts. In terms of metaphor type (abstract vs. concrete) and hemispheric processing, some researchers are examining how consumers comprehend metaphors in commercials. The study found that concrete analogies are simpler to understand than abstract ones. In hedonic product advertising, recessive metaphors outperform dominant metaphors, according to research by Bermúdez [6], although practical product promotions yielded the reverse findings. Therefore, selecting appropriate metaphor types and adjusting them to various product types and consumers’ cognitive needs can aid product designers in increasing their goods’ appeal and communication efficacy.

Symbols and metaphors find frequent application in traditional Chinese culture across various disciplines such as art [7], literature [8], and architecture [9], with art design making the most extensive use of symbols. Designers often employ conventional cultural symbols to convey their creativity and ideas in calligraphy, painting, and sculpture. For example, the terms "dragon" and "phoenix" symbolize auspiciousness, good fortune, and expectations for a brighter future. Traditional Chinese patterns like lotus, wave, and cloud motifs hold deep cultural significance and are commonly incorporated into art design. In commercial design, conventional cultural symbols are also widely employed. For brand design, traditional cultural symbol metaphors can represent the cultural implications and values associated with the brand [10]. To emphasize the grandeur and refinement of high-end brands, the iconic animals "dragon" and "phoenix" are frequently utilized in logo designs [11]. Additionally, plants such as orchids and plum blossoms, known for virtues like honesty, elegance, and purity, are commonly used in product design. These plants not only infuse items with local flavor and artistic value but also add a touch of authenticity. This paper aims to examine the creation of traditional cultural symbols from the perspectives of dominant and recessive metaphor through the study of relevant material.

### Customer experience

Chinese culture has cultivated a multitude of cultural treasures over thousands of years, many of which carry profound cultural symbolism that can be harnessed. Symbols serve as vehicles of meaning, expressing human emotions. Traditional culture encompasses essential and significant aspects that have developed within the realms of material, spiritual, and institutional spheres. Throughout history, the Chinese have pursued auspicious, contented, and fulfilling lives. Their aesthetic sensibilities and aspirations for a better existence find reflection in traditional cultural elements, which embody optimism and provide spiritual nourishment. As a result, cultural and creative products incorporating these elements offer customers diverse psychological and physical sensations. The concept of customer experience was initially introduced by Carbone and Haeckel [12]. When purchasing products or services, customers pay close attention to the overall consumption experience that the product delivers, in addition to its functional value, which includes factors such as price and quality. According to Ma et al. [13] research, customers’ emotional responses to their purchases play a significant role in influencing their purchase intentions. Kurhayadi et al.’s [14] study on the impact of customer experience on repeat purchases revealed that customers who have a positive experience are more inclined to make subsequent purchases. Godovykh and Tasci [15] investigated the process and influencing factors of the customer experience, such as emotions, perceptions, behaviors, and environments, and proposed various management solutions to enhance the customer experience. Ma et al. [16] classified customer experience into four categories based on the level of customer involvement: entertainment experience, instructional experience, escapist experience, and aesthetic experience. Gahler et al. [17] proposed an omnichannel-capable measurement of customer experience to address the lack of a common measure for various customer interactions in today’s service industries. Through seven studies, the authors develop and validate a comprehensive six-dimensional, 18-item customer experience scale, which overcomes fragmentation in existing scales and provides a consistent measure for improving customer interactions and marketing outcomes in omnichannel environments. Hoyer et al. [18] categorized customer experience into five categories based on the different functional modules of the brain: sensory experience, emotional experience, cognitive experience, behavioral experience, and relational experience.

Interacting with these cultural and creative products, customers experience a unique spiritual resonance and emotional connection, evoking a strong sense of cultural identification and belonging. In addition to captivating consumers through exquisite design and distinctive artistic expressions, cultural and creative products also engage the senses. Customers visually appreciate the fine craftsmanship and distinctive designs, experiencing visual enjoyment and appreciation. Through touch, customers feel the texture and tactile qualities of materials, fostering a sense of closeness to the products. Certain cultural and creative items employ music, sound effects, or storytelling to evoke emotions and convey narratives, stimulating customers’ auditory senses. Building upon the aforementioned research, this paper categorizes the design of customer experience for traditional cultural symbol products into three dimensions: functional experience, sensory experience, and emotional experience.

### Perceived value

Perceived value plays a significant role in consumer decision-making, influencing behaviors such as purchase intention, the amounts of purchases, and customer loyalty. The concept of perceived value was initially introduced by Kotler and Levy [19], who proposed that customers’ satisfaction and likelihood to purchase increase when they perceive a product’s value to be higher. According to other scholars, perceived value involves a compromise between the benefits consumers derive from a product and the expenses they incur, encompassing both monetary and non-monetary costs like time. Blut et al. [20] pointed out that Zeithaml defines perceived value as the consumer’s assessment of worth after considering the associated benefits and expenses. Srivastava et al. [21] argue that perceived value represents the advantages buyers believe they will receive in exchange for the costs they bear to obtain the desired benefits. Mainardes and Freitas [22] investigated the impact of perceived value dimensions on customer satisfaction and loyalty in the banking sector, comparing traditional banks and fintechs. Findings suggest that while customer satisfaction has a stronger influence on loyalty in traditional banks, fintechs excel in generating satisfaction through reliability investments. Kautish et al.’s [23] research suggests that customer perceptions of environmental value drive green purchase intentions and influence consumers’ intent to buy organic products. Paulose and Shakeel [24] investigated consumer happiness, loyalty, price sensitivity, and perceived value, finding a positive relationship between higher levels of perceived value and greater customer satisfaction. This satisfaction, in turn, fosters customer loyalty while diverting attention away from price. Subsequently, Schuir and Teuteberg [25] noted that customers make comparisons and trade-offs between perceived payoffs and received advantages, influencing their choices based on their unique circumstances. Kuppelwieser et al. [26] argued through theoretical analysis that customer perceived value is a comprehensive process response, involving customers from the beginning to the end of the purchasing journey, and is subjectively evaluated. Deepika et al. [27] investigated how switching costs affect customer loyalty by measuring both perceived value and customer satisfaction. The findings suggest that businesses aiming to build client loyalty ought to focus on satisfaction and perceived value. According to Fatmawati and Fauzan [28] research, corporate reputation fosters loyalty via trust and value, two elements that serve as key mediating variables in the model. Another important conclusion is that perceived trust has a larger impact than perceived value on customer loyalty as measured by customer satisfaction.

This paper introduces perceived value as a variable to explore the effect of cultural and creative products designed based on the metaphor of traditional cultural symbols on consumers’ purchase intention. In response to the analysis of the aforementioned literature, it is found that perceived value has a positive effect on consumers’ purchase intention.

### Cultural identity

Culture is a fundamental component that shapes the identity of a nation, ethnicity, region, and political group. Different cultures and value systems exist among various groups, distinguishing them from outsiders. In examining consumer cultural identities, Strizhakova and Coulter [29] drew attention to diverse conceptualizations and measurements of consumer cultural identity and emphasizes the necessity to further investigate the distinctions between various cultural identity categories. Depending on Von Grunebaum [30], there are three stages in the creation of cultural identity: unexplored cultural identity, explored cultural identity, and achieved cultural identity. According to Crocetti et al. [31], cultural identity is determined by three factors: (1) attachment to the heritage-cultural group and the larger society it resides in; (2) participation in practices that reflect the individual’s heritage-culture or the larger society it resides in; and (3) the individual’s and the group’s value orientation. People extend and express themselves through cultural entities, or they display themselves through cultural elements. Cultural identification partially reflects a common social psychology with good emotions. In other words, one’s cultural identification might highlight one’s good feelings about the culture to which they belong. For Chinese people who respect communal culture, cultural identity is particularly important since people frequently define themselves based on their interactions with others. Products with traditional cultural emblems are more essential to consumers in the Chinese culture, which has thousands of years of historical features. Suzhou Kunqu Opera is used as the research object by Zhang et al. [32], who also choose cultural identity as an antecedent variable. The study’s findings support the idea that a visitor’s desire to purchase goods and services is effectively influenced by their cultural identity. Zhang et al. [33] investigated the influence of consumer cultural identity and knowledge on the purchase intentions of intangible cultural heritage products (ICHP), examining the role of perceived scarcity. Results indicate that cultural identity positively affects purchase intentions, especially among consumers with higher cultural knowledge, and perceived scarcity plays a significant role. According to recent studies, there are two different ways that people respond to cultural identity: one is a reflection of their sense of national identity, which is one form of identity, and the other is a tendency for people to seek out a non-native culture that shares the same values as their own. It is obvious that traditional cultural symbol product design is a part of the identity of the source culture country. Traditional cultural symbol product design is the consumer’s identification with the country’s cultural values. According to Zong et al.’s [34] investigation into the relationship between traditional cultural symbols and cultural identity and their testing of this relationship with traditional cultural symbols, emotional value, and consumers’ purchase intentions, the results revealed that cultural identity plays a moderating role between traditional cultural symbols and consumers’ purchase intentions. Building upon the literature analysis, this study examines the role of cultural identification in consumer purchase intentions within the context of traditional cultural symbol metaphor design. It also considers cultural identification as a moderating variable between customer experience and perceived value.

## Theoretical basis and research hypothesis

### The impact of traditional cultural symbol metaphor design on customer experience

In recent years, there has been a significant focus on cultural inheritance and development at the national, social, and individual levels, resulting in a flourishing cultural and creative market. The utilization of traditional cultural symbols in product design offers a vibrant and versatile means of expression. Not only does it evoke a strong sense of beauty, but it also conveys attributes and usage information to consumers.

From a visual aesthetics standpoint, cultural symbols possess a profound ability to enhance the superficial embellishment of a product. Consumers form their initial impressions of a product based on its visual appearance, highlighting the crucial role of product design in shaping subsequent consumer perceptions [35]. The use of traditional cultural symbols in product design has the most direct and profound impact, leaving little room for misinterpretation. Furthermore, these symbols can help differentiate products in a saturated market [36]. In a world filled with mass-produced goods, products that incorporate authentic cultural elements have the power to stand out and capture attention. They act as effective marketing tools by appealing to consumers’ desire for uniqueness, personal identity, and cultural appreciation [37].

The utilization of graphics in conveying information through behavioral similarity enhances the recognizability of product functions and increases familiarity with operational behavior, giving the product an unspoken expressive function. Chinese traditional patterns encompass a wide range of colors, including common motifs like dragons, phoenixes, flowers, birds, fish, insects, mountains, and rivers. Each color holds a distinct meaning; for instance, red and purple symbolize good luck and wealth. These traditional cultural symbols, brimming with people’s well-wishes, have evolved throughout different historical periods, and each new creation brings a refreshing experience to individuals.

From the perspective of customer experience, in the era of the experience economy, design has shifted its focus to user emotions and experiences [38]. By integrating traditional elements with modern technology, animation, and other contemporary elements, product design breaks through previous monotony and boredom, boldly combining more exquisite patterns and vibrant colors, resulting in higher technological content [39]. For example, the use of traditional cultural elements in illustrative designs, as well as the introduction of audio QR codes on linen bags and other products by businesses, allows consumers to scan the code and gain insights into the product’s function, creative sources, and emotional expression. Additionally, incorporating specific music or similar means can create a pleasant shopping experience for consumers.

Traditional cultural objects hold a wealth of historical, cultural, and life-related significance. Zhao [40] classifies the metaphorical characteristics of traditional cultural symbols into modeling metaphor, material metaphor, and pattern-color metaphor. Firstly, modeling metaphor encompasses the use of lines and shapes in traditional cultural symbols to communicate cultural concepts through similarity, energy, and reference, engaging people’s imagination and associations [41]. Secondly, pattern-color metaphor involves the typical national characteristics of Chinese traditional pattern design, where color is closely linked to social status. The combination of emotional characteristics of colors and patterns reflects a certain aesthetic value [42]. Lastly, material metaphor encompasses the numerous traditional Chinese materials such as ceramics, silk, batik, and homemade cloth, which express traditional cultural concepts through unique colors and textures.

In this paper, the metaphorical design of traditional cultural products is divided into dominant and recessive metaphors. The former comprises three dimensions: modeling metaphor, material metaphor, and pattern-color metaphor, while the latter involves one dimension: symbolic culture. Building on these observations, this paper explores the impact of product appearance and customer experience from the perspective of modeling metaphor, material metaphor, and pattern-color metaphor of traditional cultural symbols in product design. Consequently, the following hypotheses are proposed:

H1: The modeling metaphor of traditional cultural symbols has a significant positive impact on customer functional experience, sensory experience and emotional experience.

H2: The material metaphor of traditional cultural symbols has a significant positive impact on customer functional experience sensory experience and emotional experience.

H3: The pattern color metaphor of traditional cultural symbols has a significant positive impact on customer functional experience, sensory experience and emotional experience.

Metaphors are a fundamental aspect of human thought processes and expressions. They establish a connection between two entities, allowing us to comprehend the unfamiliar through our familiarity with the known. Metaphorical similarity extends beyond just modeling, pattern, color, and material; it also encompasses psychological similarities rooted in culture, attitudes, and values. Hegel asserts that symbols are external objects that are immediately presented to our senses, but their true significance lies in the broader and more general meaning they imply [43]. We must distinguish between two factors when considering symbols: their meaning and the expression of that meaning. Meaning pertains to ideas and objects, regardless of their content, while representation refers to emotional states or images [44].

Drawing from symbolic anthropology, every culture can be understood as a "conceptual system of transmission expressed through a symbolic system of symbols." The symbolic system of symbols, as a system of meaning, comprises symbolic carriers, symbolic rules, and symbolic meanings. Traditional cultural symbols serve as carriers that showcase the connotation and artistic conception of a culture through products, making them increasingly popular among consumers, especially the younger generation [45]. Cultural sentiment is one of the factors driving consumer enthusiasm for locally produced goods with quality and connotation. In the marketing realm, there is a concept known as experiential marketing, which encompasses a range of activities aimed at satisfying consumers’ sensory and emotional experiences and influencing their purchase decisions [46].

In the modern market, traditional cultural elements can be incorporated into various stylistic themes such as classical, luxurious, plain, rustic, and festive, aligning with the product’s attributes and consumers’ preferences [47]. Different elements or the same elements can be expressed in diverse styles and cultural expressions. The process of extracting cultural symbols from traditional culture and attributing meaning to them can be seen as a transformation from "image" to "meaning." Traditional cultural elements possess inherent artistic value, and studies on consumers’ cognitive nervous systems indicate that strong aesthetic visual stimuli can provide a spiritual incentive to consumers. By incorporating the design of traditional cultural symbols, products can convey rich cultural connotations, infuse cultural flavor into otherwise monotonous and simple goods, and enhance their added value.

This paper argues that the symbolic meaning of traditional culture embedded in a product can enhance the customer experience and consequently promote consumers’ purchase intentions. Therefore, the following hypothesis is proposed:

H4: The symbolic cultural metaphor of traditional cultural symbols has a significant positive impact on customer functional experience.

H5: The symbolic cultural metaphor of traditional cultural symbols has a significant positive impact on customer sensory experience.

H6: The symbolic cultural metaphor of traditional cultural symbols has a significant positive impact on customer emotional experience.

### Customer experience and perceived value

Customer experience and perceived value play a crucial role in shaping consumers’ preference for a product and their willingness to make a purchase [48]. Research has shown that consumer experiences generate experiential values, including symbolic, sensory, functional, and aesthetic perceptions, which align with rational consumer values. Cultural and creative products have the ability to evoke unique emotions and sensations in customers [49]. For instance, an ancient-themed tea set can immerse customers in a rich historical ambiance and cultural significance while they savor their tea. This distinctive experience brings happiness and satisfaction to customers, thereby increasing their affinity towards the product. Similarly, a phone case inspired by a renowned painting not only showcases an exceptional design aesthetic but also offers practical protection for the phone. Customers can exhibit their taste through its usage, thereby gaining both practical and aesthetic value. This distinct perceived value elevates customers’ perception that purchasing cultural and creative products enhances their quality of life.

The value of self-direction arises from consumers’ self-evaluation, appreciation, or reflection on their consumption experience. It represents consumers’ value judgment during the consumption process [50]. A positive shopping experience significantly influences consumers’ willingness to make a purchase. Enjoyment, on the other hand, is an experiential value driven by customers’ personal preferences at the psychological level [51]. This paper argues that when consumers purchase and derive enjoyment from a product or service, the functional, sensory, and emotional experiences they encounter create positive and valuable sentiments, thereby amplifying their desire to make a purchase.

Based on the above analysis, the following hypothesis is proposed:

H7: Customers’ functional experience positively affects consumers’ perceived value of cultural and creative products.

H8: Customers’ sensory experience positively affects consumers’ perceived value of cultural and creative products.

H9: Customers’ emotional experience positively affects consumers’ perceived value of cultural and creative products.

### Perceived value and consumer purchase intention

Bajs [52] conducted a study using structural equation modeling to examine the relationship between perceived value and satisfaction. The results indicated a significant positive effect of perceived value on satisfaction. Feng et al. reviewed studies on consumer purchase intentions and found that research in this area has focused on consumer attitudes, perceived value, perceived risk, and the theory of planned behavior [53]. It has been observed that consumer attitudes can influence purchase intentions positively or negatively, and when perceived value is high or perceived risk is low, consumers are more likely to develop purchase intentions and engage in purchase behavior [54].

According to some scholars, perceived value is the subjective overall assessment made by consumers after considering the benefits and costs associated with a product or service. There is a general consensus among researchers regarding four key characteristics of consumer perceived value [55]. First, perceived value is subjective in nature, as it involves consumers’ personal perception and evaluation of a product. Second, perceived value is comparative, as consumers weigh the difference between perceived benefits and perceived costs to determine the magnitude of value [56]. Third, perceived value is hierarchical, encompassing multiple steps in the purchasing process, including product characteristics, utility, and alignment with self-expectations. Lastly, perceived value is dynamic, varying among individuals and changing in response to shifts in products and services.

Cultural and creative products elicit emotional resonance in consumers through their unique designs and creativity. These products often revolve around specific themes and incorporate cultural or artistic elements to captivate consumers’ interest and curiosity [57]. For instance, an art painting featuring a natural landscape theme can convey the beauty of nature through delicate colors and lines, evoking consumers’ yearning and desire for natural environments. This emotional resonance makes consumers feel the unique value of the product, which in turn increases their willingness to buy. Therefore, the following hypothesis is proposed:

H10: Perceived value has a significant positive impact on consumers’ purchase intention.

### Moderating role of cultural identity

It has been suggested that consumers’ affiliation with culture can directly or indirectly strengthen their sense of cultural belonging and perceived value, thereby influencing their willingness to make purchases. According to Wan and Chew [58], cultural identity refers to the psychological connection between an individual’s self and a particular culture. Another perspective emphasizes the evaluative aspect of cultural identity. Cultural identification refers to how much a person embraces particular cultural traits, as shown by their positive evaluation of the traits and values that most accurately represent a given group. Research has demonstrated that products associated with group affiliation allow consumers to establish a connection with the group and enhance their satisfaction with the product. Groups serve as channels for transmitting knowledge, beliefs, values, attitudes, traditions, and lifestyles, providing a foundation for cultural identity.

In this paper, it is argued that the cultural identity attributed to traditional cultural product symbols stems from the positive evaluation of the expressive and connotative meaning inherent in the metaphorical design of these symbolic products within the collective ideology of the nation [59]. Moreover, this cultural identity engenders emotions such as interest, pleasure, and a sense of belonging to the cultural content encapsulated in such products [60]. When consumers identify with their own country’s traditional culture, it directly heightens their perceived value of traditional cultural symbol products, thereby increasing their willingness to purchase. Additionally, this identification with their own country’s traditional culture indirectly influences their evaluation of the products, intensifying their desire to make a purchase. Therefore, the following hypothesis is proposed:

H11: Cultural identity has a positive moderating effect on the relationship between customer experience and perceived value.

**Fig 1:**
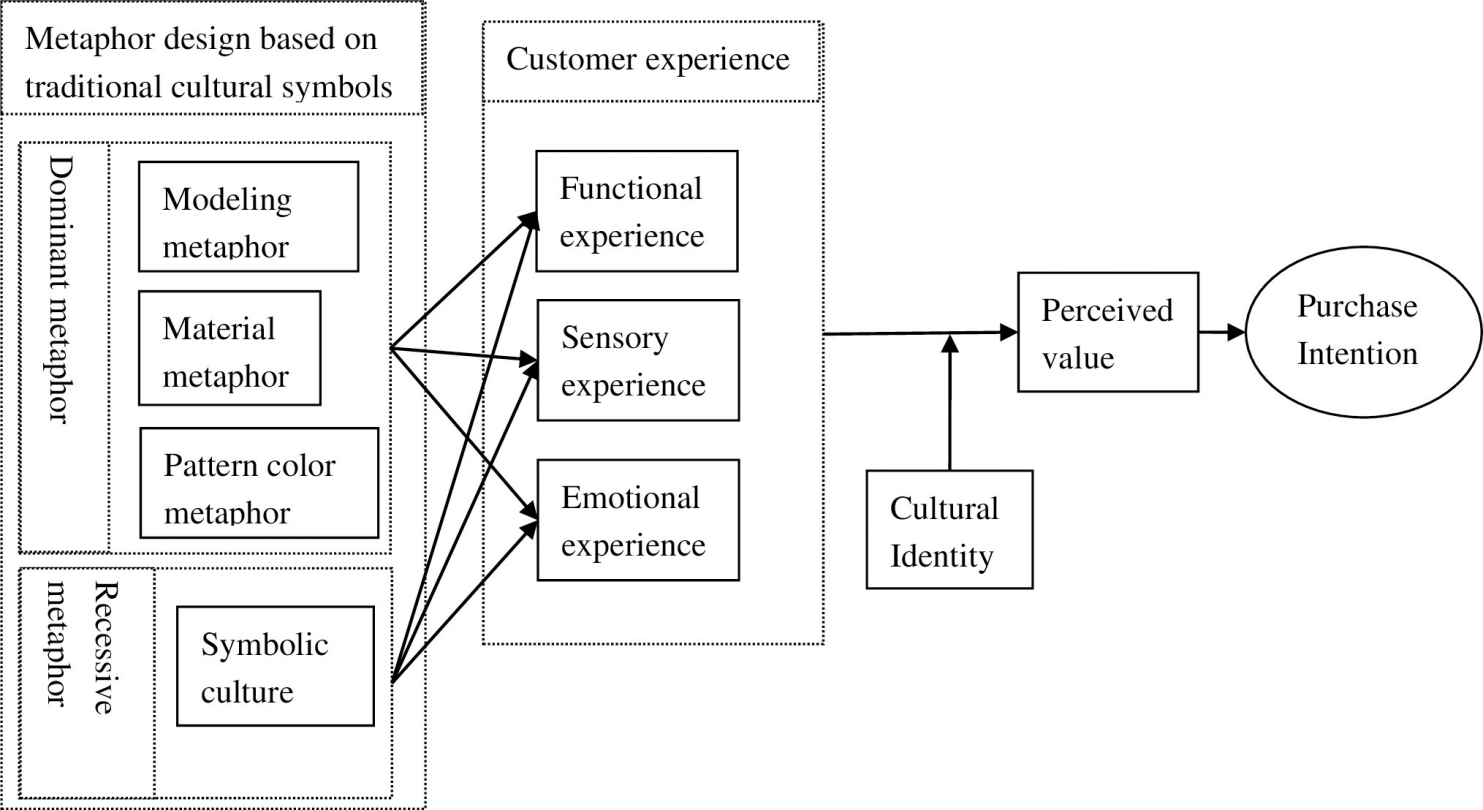
Conceptual model of traditional cultural symbol metaphor design on purchase intention.

In conclusion, this paper synthesizes relevant literature to construct a theoretical model. The key variables encompass the metaphorical design of traditional cultural symbols, customer experience, cultural identity, perceived value, and purchase intention. The theoretical model is visually represented in Fig 1:

## Research process

### Variable measurement and questionnaire design

In order to ensure a reliable and valid analysis of the questionnaire, this paper has made appropriate modifications based on the characteristics of traditional cultural symbols and has drawn from well-established scales both domestically and internationally to ensure the accuracy of the question item formulation. The questionnaire utilizes a five-level Likert scale to assess the respondents’ agreement level with each item, where 1 represents "strongly disagree," 2 represents "disagree," 3 represents "uncertain," 4 represents "agree," and 5 represents "strongly agree".

Regarding the measurement items, the metaphor design of traditional cultural symbols primarily relies on the collation and induction of recent literature. The scale has been adjusted to align with the specific characteristics of traditional cultural symbols addressed in this paper. As for customer experience, cultural identity, perceived value, and purchase intention, modifications have been made by referring to established scales in the field.

For the metaphorical design of traditional cultural symbols, this paper divides them into two categories: dominant metaphor and recessive metaphor, drawing from relevant research and the characteristics of traditional cultural symbols. The dominant metaphor encompasses three dimensions: modeling metaphor, material metaphor, and pattern color metaphor. The recessive metaphor comprises one dimension: symbolic cultural metaphor. Each dimension is measured using specific items: two for modeling metaphor, two for material metaphor, three for pattern color metaphor, and three for recessive metaphor. Based on previous scholarly work, customer experience is divided into three dimensions: functional experience, sensory experience, and emotional experience, with each dimension having three measurement items. Cultural identity is assessed through four measurement items, while perceived value consists of four measurement items derived from a comprehensive review of the literature. Regarding the study of purchase intention measurement items, the purchase intention in this paper includes four measurement items. The content of the scale in this article is shown in Table 1.

**Table 1:** Model measurement list.

Dimension	No.	Description	Source
Modeling metaphor	MO1	The shapes and lines of traditional cultural symbols can convey traditional ideas and cultural concepts	Zhao [40]
	MO2	The shape design of traditional cultural symbols enriches the formal characteristics of modern product design	
Material metaphor	MA1	Traditional materials such as ceramics and silk convey traditional ideas with their unique color, texture and texture	
	MA2	The material design of traditional cultural symbols can convey life philosophy and value orientation	
Pattern color metaphor	PA1	Traditional graphics and colors have specific cultural background and cultural implication	
	PA2	The pattern and color design of traditional cultural symbols can convey the personality characteristics of the product	
	PA3	The modeling, pattern, and material of traditional cultural symbol product design reflect changes in social structure and lifestyle	
Symbolic culture	SC1	Product design of traditional cultural symbols has an innovative combination of "tradition" and "modernity"	Simão [61]
	SC2	The myths and customs contained in the product design of traditional cultural symbols reflect the national spirit	
	SC3	The traditional craftsmanship and cultural elements used in the product design of traditional cultural symbols highlight the regional color	
Functional experience	FE1	I like the practicality of traditional cultural symbol product design	Strizhakova and Coulter [29]
	FE2	Product design of traditional cultural symbols can meet individual needs	
	FE3	Products designed with traditional cultural symbols can harvest cultural spiritual experience	
Sensory experience	SE1	Traditional cultural symbol products are attractive in terms of audiovisual and other senses	Jeon et al. [62]
	SE2	The product design of traditional cultural symbols can leave a strong sensory impression on me	
	SE3	From a sensory point of view, the design of traditional cultural symbol products is interesting	
Emotional experience	EE1	The legends and customs embodied in the product design of traditional cultural symbols can stimulate my curiosity	
	EE2	The product design of traditional cultural symbols can lead me into a certain mood or atmosphere	
	EE3	The cultural value orientation displayed by the product design of traditional cultural symbols has increased my love for it	
Cultural identity	CI1	I think the phenomenon of blindly pursuing popular elements while ignoring traditional culture is worthy of concern	Zong, et al [34]
	CI2	The products designed by traditional cultural symbols can leave a deep overall impression on me	
	CI3	I think I am similar in some way to other people whose products are designed using traditional cultural symbols	
	CI4	I think the product design of traditional cultural symbols should show traditional characteristics	
Perceived value	PV1	Relative to the cost paid, I think it is worth buying products with traditional cultural symbol designs	Lu and Wang [63]
	PV2	Relative to the effort invested, I think it is worth buying products with traditional cultural symbol designs	
	PV3	Compared with the time cost invested, I think it is worth buying products with traditional cultural symbol designs	
	PV4	Buying a product with a traditional cultural symbol design is worth more than the cost to me	
Purchase Intention	PI1	I am willing to buy products with traditional cultural symbol designs	Wang et al [64]
	PI2	I will recommend products with traditional cultural symbol designs to others	
	PI3	If there is a need in the future, I am willing to buy products designed with traditional cultural symbols	

Note: MO: modeling metaphor; MA: material metaphor; PA: pattern color metaphor; SC: symbolic cultural metaphor; FE: functional experience; SE: sensory experience; EE: emotional experience; CI: cultural identity; PV: perceived value; PI: purchase intention.

### Data collection

This study utilizes an internet-based questionnaire survey for the design and distribution of the questionnaire. The questionnaire is divided into three parts. Firstly, it collects basic personal information from the respondents, including gender, age, education, occupation, and so on. Secondly, it assesses the respondents’ familiarity with and purchase behavior related to traditional cultural symbol product design. Lastly, it measures the respondents’ perceptions across various dimensions, namely metaphor design based on traditional cultural symbols, customer experience, cultural identity, perceived value, and purchase intention.

A total of 262 questionnaires were collected through the questionnaire platform, Questionnaire Star, out of which 241 were deemed valid. Descriptive statistical analysis of the demographic characteristics of the respondents was conducted using SPSS Statistics 26.0. This analysis employed frequency and percentage indicators to gain insights into the characteristics of the research participants and facilitate a better understanding of the subsequent statistical analysis results. The findings are presented in Table 2.

**Table 2:** Sample descriptive statistics.

Measure	Item	Frequency	%
Gender	Male	99	41.08
	Female	142	58.92
Age	19~25	172	71.37
	26~30	21	8.71
	31~40	17	7.05
	41~50	13	5.39
	51~54	12	4.98
	Over 55 years old	6	2.49
Academic qualifications	High/technical secondary school and below	33	13.69
	Junior college	26	10.79
	Undergraduate	140	58.09
	Master degree and above	42	17.43
Profession	Enterprise personnel	42	17.43
	Professional technician	11	4.56
	Business and service personnel	13	5.39
	Production and transport equipment operators	9	3.73
	Student	122	50.62
	Freelancer	43	17.84
	Retirees	1	0.41
Monthly revenue	No income	113	46.89
	3000以下	30	12.45
	3000~5000	37	15.35
	5000~8000	36	14.94
	8000~10000	13	5.39
	10000以上	12	4.98
Have you ever purchased this product	Yes	201	83.4
	No	40	16.6
Are you familiar with this type of product	Yes	176	73.03
	No	65	26.97

Regarding the gender distribution, males accounted for 41.08% of the respondents, while females constituted 58.92%. This distribution aligns with the higher consumer demand among females in the cultural and creative product market, making the sample representative of the reality. In terms of age structure, 19–25 year-olds constituted 71.37%, 26–30 year-olds accounted for 8.71%, 31–40 year-olds represented 7.05%, 41–50 year-olds stood at 5.39%, 51–54 year-olds constituted 4.98%, and those aged 55 and above made up 2.49% of the sample. The majority of the sample consisted of young individuals. In relation to educational background, 13.69% of the respondents had completed high school or technical secondary education and below, 10.79% held a junior college degree, 58.09% were undergraduates, and 17.43% possessed a master’s degree or higher. This indicates a relatively high overall level of education among the respondents. In terms of monthly income, the majority reported no income, followed by less than 3000 units of currency, indicating a higher representation of middle school students and new office workers in the sample. This aligns with the characteristics of the young consumer groups who are enthusiastic about exploring new things and pursuing personalized, experiential, and hedonic consumption. Over 80% of the sample reported having purchased products with traditional cultural symbol designs, while over 70% were familiar with traditional cultural symbols, thereby meeting the survey requirements of this study. It is important to note that the participants in this study exclusively consisted of adult individuals, with no inclusion of minors. Furthermore, prior to commencing the questionnaire collection process, we provided a clear and comprehensive explanation regarding the study’s objectives and the specific utilization of the questionnaires for social science research purposes. All participants willingly and knowingly engaged in this study.

## Data analysis

This paper utilized data from a total of 241 valid samples to perform various analyses, including common method bias tests, reliability tests of measurement models, and structural model tests. The statistical software programs employed for these analyses were SPSS Statistics 26.0 and SmartPLS 3.0.

### Common method bias

Because there may be common method bias in data collected through questionnaires that affects the credibility of hypothesis testing results, this study used the Harman one-way test and potential error variable control method proposed by Podsakoff et al. [65] to test for common method bias.

According to the Harman single factor test method, using SPSS Statictics26.0 software, without rotating the factors, an exploratory factor analysis was performed on all the measurement items in this study, and 10 principal component factors with eigenvalues greater than 1 were extracted, where the largest common factor’s initial eigenvalue variance interpretation percentage is only 26.98%, which is lower than the 50% judgment standard, indicating that the explanatory power of this factor is not strong. Serious impact. It can be concluded that there is no serious problem of common method bias in the measurement process of this study.

### Measurement model testing

In this study, both reflective and constitutive constructs were employed. Reflective constructs included modeling metaphor, material metaphor, pattern color metaphor, symbolic cultural, function experience, sensory experience, emotional experience, cultural identity, perceived value, and purchase intention. On the other hand, dominant metaphor and customer experience were considered constitutive constructs. The measurement models for these constructs were evaluated based on the following test indicators.

Assessment of the reflecting measurement model’s validity and dependability Cronbach’s alpha and composite reliability scores were used to rate the constructs’ dependability. Table 3 presents the results, indicating that all constructs, namely modeling metaphor, material metaphor, pattern color metaphor, symbolic cultural, experiential function, sensory experience, emotional experience, cultural identity, perceived value, and purchase intention, exhibited satisfactory levels of reliability with Cronbach’s alpha and composite reliability values surpassing 0.7. These findings demonstrate the sound reliability of the constructs employed in this study.

**Table 3:** Reliability and aggregation validity tests.

Construct	Cronbach’s alpha	CR	AVE
MO	0.855	0.932	0.873
MA	0.874	0.941	0.888
PA	0.878	0.924	0.802
SC	0.913	0.945	0.852
FE	0.866	0.918	0.788
SE	0.858	0.914	0.779
EE	0.906	0.941	0.842
CI	0.933	0.951	0.829
PV	0.882	0.918	0.738
PI	0.837	0.902	0.755

Reliability and validity testing of the constitutive measurement model were conducted in this study. Firstly, the variance inflation factor (VIF) values of the three sub-constructs in dominant metaphor and three sub-constructs in customer experience were calculated to assess multicollinearity. Multiple regression analysis was performed using the ordinary least squares method in SmartPLS 3.0 to obtain the VIF values for the six sub-constructs, it is evident that the VIF values for the two sub-constructs are both below 3.3, indicating satisfactory scale reliability.

For the test of the validity of dominant metaphor and customer experience, the Algorithm in SmartPLS 3.0 software is used to obtain the weights of the subconstructs, and then the bootstrapping method is used to obtain the T-test value of the weights. When the T value is greater than 1.96, it indicates that the subconstruct is significant at the 0.05 level. The analysis results are that the weights of the two subconstructs reached a significant level of 0.05, which has good validity.

### Path analysis and hypothesis testing

After confirming the reliability and validity of each measurement model, in order to verify the hypothesis of the inter-influence relationship among product metaphor design, customer experience, cultural identity, perceived value and purchase intention in the theoretical model, this study tested the structural model through SmartPLS 3.0 software and tested the theoretical hypothesis proposed in this study based on the path analysis results. Table 4 shows the results of the structural model path analysis. The p-value is used to determine whether each hypothesis passes the test when determining the causal relationship between variables based on the path analysis.

**Table 4:** Path analysis results of structural model.

Hypothetical regression path	T statistics	P
MO -> FE	2.525	*
MO -> SE	2.808	**
MO -> EE	2.084	*
MA -> FE	2.372	*
MA -> SE	2.419	*
MA -> EE	2.136	*
PA -> FE	3.331	**
PA -> SE	2.355	*
PA -> EE	3.712	***
SC -> FE	1.986	*
SC -> SE	3.034	**
SC -> EE	4.575	***
FE -> PV	3.014	**
SE -> PV	4.248	***
EE -> PV	3.196	**
PV -> PI	9.469	***
CI×FE -> PV	2.587	*
CI×SE -> PV	0.636	0.524
CI×EE -> PV	0.113	0.910

Significance level

*10%

**5%

***1%

From the results of the path analysis, it was found that these were hypothesized to be supported in the direct effect hypothesis. Cultural identity has a positive moderating effect between functional experience and perceived value in customer experience (p<0.05), but the moderating effect between sensory experience and emotional experience and perceived value by cultural identity is not significant, so there is no significant moderating effect between cultural identity and customer experience and perceived value. The results of the hypothesis testing are summarized in Table 5.

**Table 5:** Test results of direct effect hypothesis.

Hypothetical regression path	Research hypothesis	Supported
H1	MO -> FE	yes
	MO -> SE	yes
	MO -> EE	yes
H2	MA -> FE	yes
	MA -> SE	yes
	MA -> EE	yes
H3	PA -> FE	yes
	PA -> SE	yes
	PA -> EE	yes
H4	SC -> FE	yes
H5	SC -> SE	yes
H6	SC -> EE	yes
H7	FE -> PV	yes
H8	SE -> PV	yes
H9	EE -> PV	yes
H10	PV -> PI	yes
H11	CI×FE -> PV	yes
	CI×FE -> PV	no
	CI×FE -> PV	no

Combined with Tables 4 and 5, the results of the direct effect hypothesis test of this study are discussed as follows:

1. The results of this study indicate that traditional cultural symbol metaphor design has a significant positive impact on customer experience, supporting hypotheses H1 to H6. The metaphorical design of traditional cultural symbol products emphasizes the communication of cultural values and incorporates consumers’ expectations through design techniques. This emotional resonance with consumers enhances the recognition of these products compared to similar ones. As a result, it leads to diverse functional experiences, sensory experiences, and emotional experiences for customers.
2. The findings also demonstrate that customer experience has a significant positive effect on perceived value, supporting hypotheses H7 to H9. A positive customer experience encompasses factors such as convenient shopping, friendly customer service, and high-quality products or services. When customers have a favorable experience, they perceive the actual value of cultural and creative products to be higher than their psychological value. Thus, a great customer experience plays a crucial role in the long-term success of a brand or company, contributing to customers’ perceived value of the product or service.
3. Furthermore, perceived value has a significant impact on consumers’ willingness to purchase (p<0.001), supporting hypothesis H10. Consumers’ purchasing decisions are driven by maximizing their benefits and considering the costs involved. The level of perceived value directly influences consumer satisfaction. When consumers perceive high value, their post-purchase satisfaction increases. On the contrary, if the perceived value is low, post-purchase dissatisfaction may diminish their willingness to make future purchases. Therefore, perceived value plays a significant role in consumers’ purchase decisions as it directly relates to their desire to maximize benefits and their satisfaction after the purchase.

The moderating impact is discussed in the material that follows. The results of the moderator effect analysis reveal that cultural identity plays a significant role in moderating the relationship between functional experience and perceived value in customer experience. Cultural identity refers to the shared values and beliefs of customers associated with a particular cultural background or community. When a product or service aligns with a customer’s cultural identity, they are more likely to perceive it as consistent with their own values. This alignment enhances their sense of identity and satisfaction with the functional experience offered by the product or service. Cultural and creative products often incorporate symbols and signs that hold shared meanings within a specific culture. When these symbols and signs resonate with a customer’s cultural identity, it fosters emotional connection and amplifies the perceived value of the product or service.

On the other hand, the analysis indicates that the moderating effect of cultural identity on the relationship between sensory and emotional experiences and perceived value is not significant. This could be attributed to individual differences. While cultural identity may exert some influence on sensory and emotional experiences, each person’s sensory preferences and emotional encounters are unique. Hence, the moderating effect of cultural identity on sensory and emotional experiences tends to be relatively limited.

## Discussion

This paper aims to investigate the utilization of traditional cultural symbols in product design through empirical analysis. Firstly, this paper examines the impact of traditional cultural symbols on customer experience, perceived value, and purchase intention, focusing on product metaphors such as shape, material, pattern and color, appearance, and symbolic culture. Furthermore, it analyzes the moderating role of cultural identity in the relationship between customer experience and perceived value, yielding the following specific findings:

1. Traditional cultural symbols, including their modeling, material, and pattern color metaphors, positively impact customers’ functional, sensory, and emotional experiences. Modeling metaphors, (such as the traditional Chinese "fortune" character decoration) convey meanings of auspiciousness and happiness through specific shapes and designs, Which not only enhances customers’ sense of identification and familiarity with the product or venue but also improves functional experiences and reinforces trust in the product. Then, material metaphors involve the selection of culturally relevant materials, such as the use of red in traditional Chinese culture to symbolize joy and auspiciousness. Not only do they enhance the perceived quality and durability of the product, but they also elevate functional experiences. Additionally, material metaphors enhance sensory experiences through tactile sensations and material qualities, while evoking emotional resonance, thereby enriching emotional experiences. Lastly, pattern and color metaphors involve the selection of specific patterns and colors to convey symbolic meanings of cultural symbols, such as traditional Chinese dragon and phoenix motifs and the color red. they enhance product attractiveness, distinctiveness, and functional experiences, while also heightening sensory experiences through visual pleasure or excitement. Moreover, pattern and color metaphors foster emotional resonance, further enhancing emotional experiences. Overall, the modeling, material, and pattern color metaphors of traditional cultural symbols positively influence customers’ experiences, enhancing functional, sensory, and emotional dimensions, and thereby supporting businesses in shaping product images and strengthening brand identity.
2. Traditional cultural symbols’ symbolic cultural metaphors exert a significant positive influence on customers’ functional, sensory, and emotional experiences. They represent profound historical and cultural connotations and are widely employed in commercial settings. Through the utilization of symbols, customers develop a sense of belonging and pride, enhancing their emotional experiences. Simultaneously, the selection of traditional cultural symbols conveys specific brand images and value propositions, bolstering customers’ trust and identification, thereby enhancing functional experiences. Prior research may have predominantly focused on one or two aspects, such as emotional experiences, while neglecting functional and sensory experiences. The distinctive feature of this study lies in its comprehensive and meticulous analysis of the impact of traditional cultural symbols, revealing their positive effects across multiple dimensions of customer experience. This offers deeper theoretical support for businesses aiming to leverage traditional cultural symbols in product design and marketing strategies.
3. Functional, sensory, and emotional experiences within customer interactions significantly influence perceived value. This conclusion is drawn from a multidimensional analysis of customer experiences, encompassing functional, sensory, and emotional aspects. Functional experience pertains to the utilitarian satisfaction derived from product or service usage. For instance, an intuitive and efficient smartphone application enhances users’ functional experience, thereby increasing perceived value. Sensory experience involves sensations perceived through sensory organs such as vision, audition, and touch. For example, a meticulously designed restaurant that offers delicious cuisine, a comfortable ambiance, and pleasant music enhances customers’ sensory experience, consequently elevating perceived value. Lastly, emotional experience refers to affective responses elicited by products or services. For instance, a company with a core environmental ethos, manifested through sustainable products and services, fosters emotional resonance among customers, thereby increasing perceived value. In comparison with prior research, this conclusion provides a more comprehensive consideration of the diverse dimensions of customer experience in influencing perceived value. By comprehensively examining functional, sensory, and emotional experiences, this study elucidates their collective impact on perceived value, offering nuanced theoretical underpinnings for businesses to effectively enhance the perceived value of their products or services.
4. Perceived value exerts a significant positive influence on consumer purchase intention. This is because as consumers perceive higher value in a product or service, they are more likely to deem it worthy of purchase, thereby enhancing their inclination to make a purchase. Perceived value encompasses consumers’ comprehensive evaluation of the benefits and costs associated with a product or service, including factors such as product quality, performance, price, and brand reputation. Consequently, when consumers perceive the value of a product or service to outweigh its costs, they are more inclined to choose to purchase, aligning with both common sense and economic theory. This finding is consistent with previous research results, which have extensively demonstrated the significant impact of perceived value on consumer purchase intention. For example, some scholars have proposed the Value-Satisfaction-Loyalty chain model in their seminal research, emphasizing the influence of perceived value on consumer satisfaction and loyalty. Other studies have similarly found that higher perceived value leads to increased propensity for purchase and repurchase. Therefore, the importance of the validation result lies in its emphasis on the critical role of perceived value in consumer purchase decision-making, which is consistent with previous research findings. The further bolsters confidence and reliability in the theory.
5. Cultural identity exerts a positive moderating effect on the relationship between functional experience and perceived value within customer experiences. Cultural identity refers to an individual’s degree of identification with their cultural group, including alignment with cultural values, beliefs, and traditions. Research indicates that when customers perceive a high degree of alignment between the cultural symbols, values, and traditions conveyed by a product or service, their functional experience and perceived value are enhanced. Firstly, cultural identity enhances functional experience. When customers perceive a high level of consistency between the cultural symbols and values of a product or service, they are more likely to comprehend and accept its functional characteristics, thus becoming more willing to use and enjoy the functional experience it provides. For example, a company that integrates traditional cultural elements into product design, such as traditional Chinese red and dragon-phoenix motifs, can attract the attention of Chinese consumers and enhance their sense of functional alignment with the product, thereby enhancing functional experience. Secondly, cultural identity also positively influences perceived value. When customers resonate with the cultural symbols and values conveyed by a product or service, they experience a heightened emotional connection and sense of identity with the brand, thereby strengthening their perceived value of the product or service. In contrast with previous research, this study emphasizes the moderating role of cultural identity in the relationship between functional experience and perceived value. Prior research may have predominantly focused on the influence of product or service characteristics on customer experience, overlooking the moderating effect of cultural factors. The findings of this study provide a deeper understanding for businesses, guiding them to better utilize cultural identity in product design and marketing strategies to enhance the relationship between functional experience and perceived value.

## Management implications and prospect

### Management implications and theoretical implication

In the study exploring the impact of traditional cultural symbols on consumers’ willingness to purchase in product metaphor design, it has been observed that the modelling metaphor, pattern and color metaphor, material metaphor, and symbolic culture metaphor employed in the design of traditional cultural symbols products have a positive influence on customer experience. This suggests that with the growing emphasis on traditional cultural heritage in recent years, people are increasingly open to products associated with traditional culture. They seek cultural connotations embedded in products and place greater importance on the overall product or service experience. In light of these findings, this paper proposes several management insights to provide guidance for enterprises:

First, incorporating traditional cultural symbols into product design can enhance expression. The research findings demonstrate that traditional cultural product design enables consumers to derive functional, sensory, and emotional experiences. Thus, companies should consider integrating traditional cultural symbols or elements into their product designs, leveraging historical, ethnic, and traditional aspects of traditional culture. Designers should strive to establish emotional resonance between consumers and products based on their own cultural values. Additionally, they should enrich the expression of traditional cultural elements in terms of modeling, materials, patterns, colors, and cultural symbolic representations.

Second, emphasizing product quality and enhancing user experience is crucial. The research findings indicate that customer experience significantly influences consumers’ purchase intentions. As traditional cultural products are still commodities, enterprises should prioritize product quality in their design processes. This includes aspects such as refined aesthetics, practicality, and durability. Through market research, companies should gain an understanding of users’ preferences to create high-quality products that offer good value at an affordable price, thereby building a solid corporate reputation. In product marketing, enterprises can explore the use of technology, such as augmented reality (AR), virtual reality (VR), and other virtual reality technologies, to present the appearance and essence of products in novel ways. This approach can arouse consumers’ curiosity and excitement, effectively convey product information, and enhance consumers’ overall consumption experience.

Third, focusing on cultural values and leveraging the role of products as cultural heritage are essential. Although there is no significant moderating effect of cultural identity between customer experience and perceived value, cultural identity does exert a positive influence on perceived value. Hence, when designing products, companies should center their efforts on the cultural value of traditional culture. By conducting user research and other means, they should gauge consumers’ familiarity with traditional cultural symbols. It is advisable to incorporate widely recognized traditional cultural symbols, which can serve as a foundation for enriching the expression of traditional culture in products. Moreover, enterprises should embrace the responsibility of cultural heritage preservation. They should unearth the outstanding aspects of traditional culture and identify traditional cultural symbols worthy of promotion. By applying them to product design, companies can facilitate broader awareness and appreciation of Chinese traditional culture.

These proposed management inspirations aim to provide valuable guidance to enterprises operating in the realm of traditional cultural symbol products, assisting them in leveraging the rich cultural heritage and delivering enhanced consumer experiences.

Theoretical significance encompasses three primary aspects:

First, this study contributes to the theoretical framework of metaphor design mechanisms involving traditional cultural symbols. It adds richness to the theoretical landscape within the cultural creative product design domain, offering profound insights into the role of traditional cultural symbols in shaping product design.

Second, the application of empirical analysis methods enables a systematic examination of existing research theories, addressing their limitations. Through the incorporation of relevant theories on product metaphors, this study bridges gaps in empirical analyses from previous research, thereby enhancing the theoretical depth of the influence mechanism associated with traditional cultural symbol product design.

Lastly, the empirical findings of this study lend support to the validation of metaphorical patterns and design rationality. Moreover, they furnish a new theoretical foundation and practical recommendations for the implementation of traditional cultural symbol design methodologies in the preservation and advancement of traditional culture, as well as in the development and design of products.

### Limitation and prospect

The paper exhibits certain limitations, which are summarized as follows:

1. Limitations of Measuring Tools:
  Due to the relatively nascent nature of the cultural and creative industry as a research field, especially in terms of quantitative research, there is a scarcity of relevant literature. Consequently, the study relies on building models and designing questionnaires based on theoretical foundations and referencing research from related industries, rendering it more of an exploratory nature. The verification of predicted hypotheses in the final model remains inconclusive. While the questionnaire’s reliability is commendable, the influencing factors proposed lack substantial innovation and largely draw from prior studies.
2. Limitations of Research Objects:
  The distribution of the questionnaire via the internet results in a predominantly young and internet-active respondent base, with 80.08% of participants being under the age of 30. While this aligns with the demographic of cultural and creative consumers, it fails to fully capture the perspectives of consumers in other age groups, thus limiting the generalizability of the findings.
3. Limitations of Variable Settings:
  The variable settings in this study are grounded in customer experience and perceived value theory. Despite extracting multiple variables, the consideration is not exhaustive, omitting factors such as emotional and environmental elements. This oversight restricts the comprehensiveness of the study’s conclusions.

The future research prospects for this paper are outlined below. As cultural endeavors continue to evolve, the cultural and creative industry is poised for broader market expansion and developmental opportunities. Enhancements to future research can be considered in the following dimensions:

1. Expand Sample Size and Enhance Quality:
  The current study’s valid sample size is 241, exclusively sourced from the internet. Future research can adopt a dual approach, involving both offline and online questionnaire distribution, with an expanded sample size and broader age range. Given the distinct environments of online and offline shopping, a comparative analysis could be conducted by establishing a comparison group. Rigorous screening of collected data should be performed to eliminate invalid questionnaires and ensure the reliability of results.
2. Explore Additional Factors and Incorporate New Variables:
  Consumer behavior is intricate, influenced by various factors that may evolve over time. Subsequent research should delve into a more comprehensive exploration of factors impacting consumer attitudes and intentions, allowing for the refinement and enrichment of the existing model.
